# A Computer Simulator for Assessing Different Challenges and Strategies of *de Novo* Sequence Assembly

**DOI:** 10.3390/genes1020263

**Published:** 2010-09-13

**Authors:** Bjarne Knudsen, Roald Forsberg, Michael M. Miyamoto

**Affiliations:** 1 CLC bio, 8200 Aarhus N, Denmark; E-Mails: bknudsen@clcbio.com (B.K.); rforsberg@clcbio.com (R.F.); 2 Department of Biology, Box 118525, University of Florida, Gainesville, Florida, 32611-8525, USA

**Keywords:** assembly simulator, read simulator, *de novo* assembly, sequencing strategies, next generation sequencing

## Abstract

This study presents a new computer program for assessing the effects of different factors and sequencing strategies on *de novo* sequence assembly. The program uses reads from actual sequencing studies or from simulations with a reference genome that may also be real or simulated. The simulated reads can be created with our read simulator. They can be of differing length and coverage, consist of paired reads with varying distance, and include sequencing errors such as color space miscalls to imitate SOLiD data. The simulated or real reads are mapped to their reference genome and our assembly simulator is then used to obtain optimal assemblies that are limited only by the distribution of repeats. By way of this mapping, the assembly simulator determines which contigs are theoretically possible, or conversely (and perhaps more importantly), which are not. We illustrate the application and utility of our new simulation tools with several experiments that test the effects of genome complexity (repeats), read length and coverage, word size in De Bruijn graph assembly, and alternative sequencing strategies (e.g., BAC pooling) on sequence assemblies. These experiments highlight just some of the uses of our simulators in the experimental design of sequencing projects and in the further development of assembly algorithms.

## 1. Introduction

**Figure 1:**
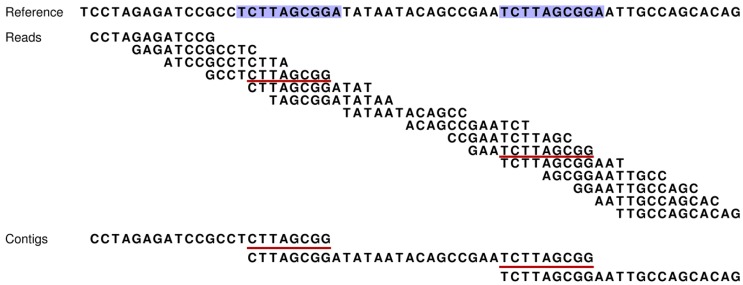
How our assembly simulator works to determine which contigs are theoretically possible (and conversely, which are not). At the top, a short stretch of sequence is presented for a reference genome [3]. This short region includes two direct repeats (blue shading). Fifteen reads (without sequencing errors) are given for this region and are ordered according to their overlapping sequences. However, the occurrence of the two repeats introduces ambiguities that make this ordering ambiguous (e.g., contig one could be directly linked to either contig two or three). Such ambiguities result in contig breakpoints, and thereby, increased numbers of shorter contigs. Our assembly simulator recognizes these breakpoints as those overlaps of successive reads (red underlining), which occur multiple times in the reference (blue).

When sequencing long strands of DNA, the dominant approach is to use a form of “shotgun sequencing” where the DNA is broken randomly into smaller fragments from which reads of the DNA sequence can be experimentally produced [1]. Since several sequencing passes are made on the molecule, reads will partially overlap and computer programs can use this information to infer the original sequence through the process of “*de novo* assembly” [2]. The lengths of typical reads vary from about 35 bp to about 1000 bp depending on the sequencing technology used. *De novo* assembly can be very difficult, especially due to experimental sequencing errors and the repeated sequence regions that are present in most genomes. If a repeated region is longer than the read length, it is not immediately possible to obtain the complete original sequence by *de novo* assembly since the overlap information is ambiguous (see Figure 1). Hence, the goal is to infer the longest possible subsequences of the original (contigs) while not making any errors. 

A number of factors will affect the price and quality of a sequencing result and the researcher undertaking the experiment must balance these to get an acceptable end result. There are many different sequencing technologies available with the most common ones being dideoxynucleotide chain-termination or “Sanger” sequencing [4,5], pyrosequencing as supplied by the company 454, a subsidiary of Roche [6], reversible terminator sequencing as provided by Illumina [7], and ligation-base sequencing as supplied by Applied Biosystem through the SOLiD system [8]. These technologies differ in the price per sequenced base, in the length of the reads and in their error profiles, and in the amount of data produced. Each technology has unique advantages and disadvantages and it is desirable to select a technology, or combination of technologies, that is suitable for the complexity of the genome being sequenced (for reviews, see [9,10,11]).

An important parameter to decide for the experiment is read coverage or sampling depth of the genome [1,2]. At low coverage, regions will occur where no or too few reads are present to ensure sufficient data to infer a continuous sequence. As coverage increases, the probability of observing such insufficiently sampled regions decreases but this also increases the cost of the experiment.

For all sequencing technologies, there is also the option of using a range of paired end sequencing protocols which creates reads in pairs that are known to originate from a certain distance from each other in the original sequence [2,12,13]. Errors do, of course, occur, so reads thought to form pairs may indeed not be paired, and the specific distance is subject to some uncertainty. Nonetheless, paired end reads (paired reads or mate pairs) provide a good way to bridge repeated regions and can improve *de novo* assembly results significantly by joining subsequences into longer scaffolds. The improvements gained from using paired end information will, however, depend on the specific protocols used and how well the choice of the mate pair distance(s) matches the repeat structure of the sequenced genome.

A choice also exists as to whether shotgun sequencing should be performed on the whole genome at once or through hierarchical approaches where the genome is first divided into smaller parts that are then shotgun sequenced and *de novo* assembled independently to reduce the complexity of the analysis [1]. At the highest level, subdivision can be accomplished by separating individual chromosomes by flow cytometric sorting prior to sequencing and assembly [14]. Alternatively (or in combination), the genome can be subdivided to a much larger degree by cloning of large chromosomal fragments into bacterial artificial chromosomes (BACs) or yeast artificial chromosomes (YACs) [1,15]. As a hierarchical approach adds a significant cost to a sequencing project, a decision must be made as to how great an improvement in the end result can be expected as compared to the less expensive, whole genome shotgun sequencing approach.

Once an experimental result is produced, a decision must also be made regarding the use of *de novo* assembly software. Recent advances in sequencing technologies have sparked the development of a new generation of *de novo* assembly programs. These include Velvet [16], SOAPdenovo [17], ABySS [18], ALLPATHS [19], and the *de novo* assembler from CLC bio [20], which all use a so-called De Bruijn graph approach [21]. These programs each have unique strengths and weaknesses that depend on the chosen technology and sequencing strategy. A common feature of the programs is the possibility to vary the word size upon which the construction of the De Bruijn graph is based (e.g., see [22]). However, due to the computational cost of large assembly jobs, it is often not possible, or desirable, to exhaustively try all possible word sizes and it is therefore important to guide exploration to the most relevant part of the parameter space.

In the completion of a *de novo* sequencing experiment, a researcher must address all of the options listed above and make the relevant choices. Often, there will be nothing else than common practice, previous experience, and educated guesses to guide this decision.

**Figure 2:**
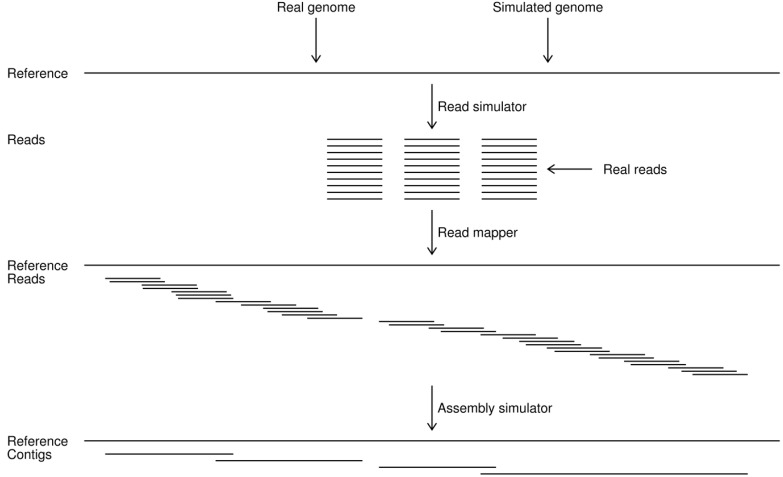
Work flow in the assembly simulations. A reference genome is first chosen as either a known sequence or is simulated from a previously determined one. Reads are then simulated from the reference or taken as a set of real reads for the known genome. The simulated or real reads are mapped to the reference and a set of simulated contigs is generated by the assembly simulator. This study provides the read and assembly simulators. The read mappings in this paper were done with the CLC Assembly Cell programs [24], but can be accomplished with any standard read mapping software (e.g., [25,26,27,28]).

To assist a researcher in making these choices on a more rational foundation, we here describe a newly developed program which simulates an assembly: It gives an estimate of the best possible *de novo* assembly result from a given data set [23]. The program we provide is not a *de novo* assembly program, but instead it assumes that the original sequence (reference) is actually known, and based on that information is able to identify what result is theoretically possible from a given set of reads (Figure 2). This program can be used when selecting shotgun sequencing strategies, paired end protocols, coverage depth, and the like, and when developing and evaluating new *de novo* assembly algorithms.

## 2. Read and Assembly Simulators

### 2.1. Read Simulator

Our read simulator can generate any number of reads of a predetermined length from the reference genome (Figure 2). Their number can either be designated explicitly or in terms of genome coverage. The simulator can randomly generate reads from both strands of the reference. Paired reads can be simulated by designating the relative orientations and range of the intervening distances between their ends. The final distances are randomly chosen from a normal distribution such that 95% of the pairs fall within the specified range. The read simulator can also introduce sequencing errors, including nucleotide differences, insertions, and deletions. For example, it can generate reads with color space errors to imitate SOLiD data.

### 2.2. Assembly Simulator

Our assembly simulator is the more novel component of our new computer program (Figure 2). This simulator relies on the reference genome to determine what *de novo* assembly is optimally possible for the available reads. It works on the read mappings to determine which regions cannot be connected into contigs given the sequence information. The basic idea is that if the overlapping sequence of two successive reads falls within a repeat, and thereby occurs more than once within the reference, then these reads cannot be unambiguously assembled into a single contig (Figure 1).

Since our focus is on the contigs, our assembly simulator ignores minor errors in the reads (e.g., small mistakes that result in single nucleotide differences or short deletions and insertions) and concentrates instead on only their mappings to the different regions of the reference. If part of a read matches very poorly, then it is not mapped by the assembly algorithm to its region of the reference. In this way, read subsequences (or whole reads) with larger errors are ignored. This handling of sequencing errors works well, except in certain exceptional instances where the optimal contig lengths can become overestimated (e.g., due to repeated biased mistakes at the same sites of different reads).

The assembly simulator first sorts the reads according to their starting positions in the reference and then processes them in that order. A read that ends before that of the previous one is discarded, since it constitutes a subsequence of the latter and thereby contributes nothing to the assembly. Thus, the final list of reads includes only those with both increasing end as well as start positions. After such sorting and processing, the assembly simulator continues with the comparison of the overlaps between successive reads. 

In terms of *de novo* assembly, repeats are simply sequences that occur more than once in the genome. Thus, repeats need not belong to a known family of sequences nor consist of sequences that are necessarily common in the genome [29]. Our assembly simulator does not form contigs in the absence of reads spanning a repeat and thus no contig will carry this sequence. This handling of a longer repeat (*i.e.*, one longer than the reads) differs from that of most *de novo* assembly programs, which attempt to output one copy of each such sequence. Conversely, a short repeat whose two flanking sequences occur within a single read is included within a contig. Thus, multiple dispersed copies of a shorter repeat will often be found among several different contigs.

### 2.3. Assembly Simulations withPaired Reads

Our assembly simulations treat each paired read as a single long read that stretches from the start of the first mate to the end of the second one [31]. Consider the simplest case of a single paired read. When simulating the assembly for this pair, a single long contig that encompasses the two mates and their intervening sequence is obtained, provided that the end distance falls within specified limits. In short, the result is a scaffold with an unknown intervening sequence between the two sequenced ends. In this study, we generally use the term “contig” to denote any sequence region that is connected either by overlapping reads or mate pairs. Thus, we do not make a strong distinction between contig and scaffold, since the two are treated very similarly. One can argue that this result is too optimistic, since nothing is known about the intervening sequence between the two ends. However, we do know the relative location of the ends, albeit with some uncertainty about their actual distance.

**Figure 3:**
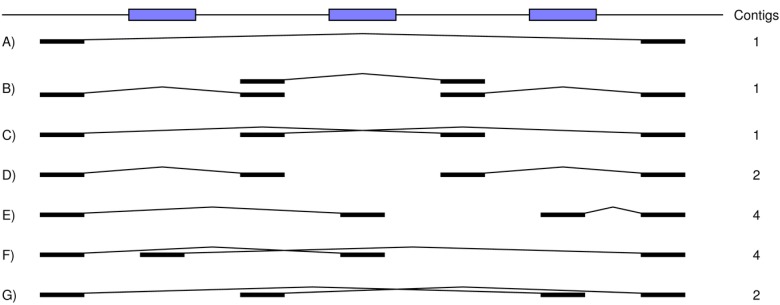
How our assembly simulator treats paired reads as single long reads. The reference sequence (top) includes three repeats (blue boxes). The thin lines in A) to G) interconnect the two ends of a pair. It is assumed that sufficient read coverage exists to connect the intervening non-repeat regions between the ends of each pair. A) One contig is assembled as the single pair spans all three repeats. B) Again, one contig results as each of the three pairs spans one of the three different repeats. C) Once more, one contig is formed as the two pairs each span two of the three different repeats. D) In contrast, two contigs are now assembled as the second repeat is not spanned. E) Both pairs are discarded, since one read of each occurs within a repeat and therefore cannot be uniquely mapped to the reference. Thus, four contigs are assembled for the same reason as illustrated in Figure 1. F) Four contigs are also formed for the same reason as in E). G) The first pair, but not the second, is discarded, since its second read occurs within a repeat. Thus, two contigs are assembled with their breakpoint occurring in the first repeat.

To be included in the assembly simulations, both ends of a paired read must be uniquely mapped to the reference. Thus, repeats once again limit the assembly (Figure 3). This use of the paired reads works well, provided that a good baseline of sequence coverage (e.g., 15-20 times depth; see below) exists to ensure that the separate contigs are well defined. With a good baseline of coverage, the paired reads interconnect the shorter contigs into longer ones.

### 2.4. Assembly Simulations forPhase Determination

**Figure 4:**
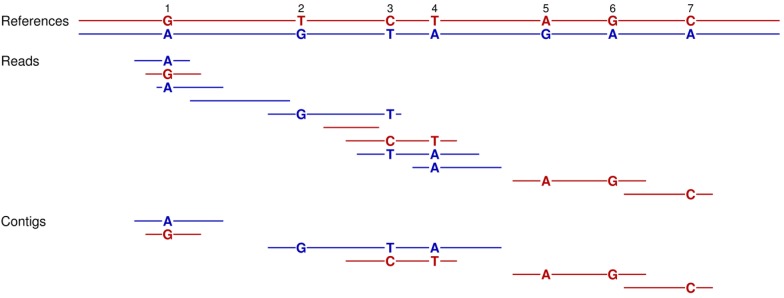
How our assembly simulator can determine the phase of the linked heterozygous sites from the reads for a diploid individual. A standard assembly simulation is performed for the 11 reads of the red *versus* blue homologous references of a single diploid. Those reads that do not cover any of the seven polymorphic sites are discarded, since they can be mapped to either the red or blue reference (e.g., reads four and six). Similarly, those reads with overlaps that do not include a polymorphic site are left unconnected, since their overlapping sequences can be mapped to either the red or blue reference (e.g., the last two reads). In contrast, the other reads with overlapping polymorphisms can be assembled into contigs, since their overlaps can be mapped by their different heterozygous bases to either the red or blue homolog, but not both (e.g., reads one and three). The final contigs allow for the determination of phase (haplotypes) for heterozygous sites 2, 3, and 4 and for positions 5 and 6.

Sequence assemblies for diploid individuals typically focus on the generation of contigs for one, rather than both of the homologs, of their two chromosome sets [32,33]. Then, read mapping is conducted to identify which sites are heterozygous. In some cases, one can also determine the phase of the base differences at neighboring heterozygous sites (*i.e.*, the haplotypes) [34,35]. Such phase determination is most likely when the linked heterozygous positions are close enough to be included in a single read and/or when the reads are sufficiently long to provide information about these neighboring polymorphic sites [36]. A special application of the assembly simulator is that it can be employed to evaluate when the neighboring heterozygous sites are close enough and/or the reads are sufficiently long for phase determination. Where the two copies are homozygous, they are identical and the assembly simulation correspondingly treats them as repeat regions. Thus, no contigs are assembled for these homozygous regions. In contrast, if base differences (or small insertions or deletions) occur at neighboring heterozygous sites, then the two copies differ and assembly of the reads into contigs becomes possible (Figure 4). Phase determination then becomes a matter of examining the assembled contigs to see which different bases at heterozygous sites are linked as haplotypes.

### 2.5. SimulatingWord Size Effects in De Bruijn Graphs

Another interesting application of our simulation tools is to test for the effects of different word sizes on sequence assemblies by De Bruijn graph methods [2]. These effects can be evaluated by incorporating single base differences as random sequencing errors into the simulated reads. An optimal assembly for these reads is obtained with our assembly simulator and is then compared to those from a De Bruijn graph approach to assess the effect of word size on the performance of the latter.

**Figure 5:**
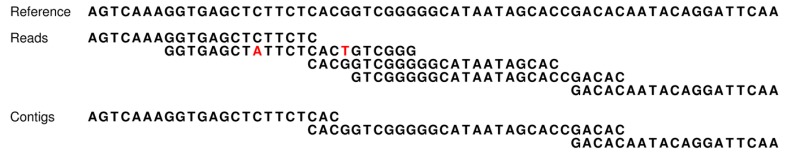
How word size can influence the performance of a De Bruijn graph assembly. Five reads are compared with the word size set to seven. The second read includes two miscalled bases as highlighted in red. The overlap between the last two reads is only five bases long and is therefore too short given the word size of seven. Thus, the last read is left unconnected as its own contig. In turn, the six bases after the second error of the second read are ignored, since no word of length seven exists after this mistake. Thus, two separate contigs are formed at this point, since the effective overlap between the second and third reads is only three bases (CAC, which occurs three times in the reference and is also shorter than the six nucleotide overlap required for a connection). In contrast, the eight bases upstream of the first error in the second read are retained, since they include words of length seven. Correspondingly, the first two reads are connected into a single contig by their overlapping words prior to the first error.

De Bruijn graph algorithms are usually at least somewhat sensitive to the word size chosen for the assembly. If the chosen word size is too short, then the graph becomes very complicated and the assembly is usually poor. In turn, if the word size is too long, then some other effects can reduce the assembly quality for reasons that are highlighted in Figure 5 and below.

The De Bruijn graph approach begins with the generation of a large table that includes all of the words of a given size, which are present in the reads. A word is then selected from the table and a DNA base is added to its end, while its first letter (nucleotide) is removed from its start. Given the four DNA bases, one word is thereby transformed into four others that may or may not occur within the table. If one of the four words is represented, then this word and its predecessor are connected together as a small contig. This process is continued until either no additional connections exist or two or more such links are encountered. Thus, in the formation of a contig, a word and its unique neighbor must occur in two different reads such that their overlap length is at least one less than the word size (Figure 5). If the overlap is shorter, then the contig is broken at this point, just like when it is present twice in the reference. This dependency on a minimal overlap highlights one way in which long word sizes can limit a De Bruijn graph assembly. 

A related problem with long word sizes is that a single sequencing error of a read affects many neighboring words (Figure 5). For example, if the word size is set to 20 and position 15 of a read contains an error, then its first 15 words will differ from their correct subsequences in the reference. In effect, the first 15 nucleotides of this read are discarded by the De Bruijn graph algorithm, thereby making it more difficult to connect reads into contigs by their minimal overlaps [2]. Our read and assembly simulators can model both of these effects on a De Bruijn graph approach by introducing sequencing errors and by invoking its word size option, respectively.

## 3. Computer Simulations

Here, we present various applications of our new simulation tools, which highlight just some of their many uses in the evaluation and design of different sequencing and assembly strategies. Some of these simulations relied on the whole human genome as the reference (Human Genome Reference DNA Sequence, build 36.1 from NCBI). This reference genome is 3080 Mbp in length, with 222 Mbp (7.21%) consisting of unknown nucleotides (symbol “N”). Many of these unknown nucleotides occur in blocks of at least 10 or 100 kbp (265 and 84 such regions, respectively, with the longest spanning 30 Mbp). For other simulations, a smaller, more convenient, yet fully instructive dataset was used. Chromosome 2 was selected for these simulations, since it is still relatively long (243 Mbp) and captures much of the complexity of the entire human genome, but with fewer unknown regions (2.16%). Specifically, it contains 17 and nine unknown regions with lengths of at least 10 kbp and 100 kbp, respectively. In contrast, chromosome 1 (for example) is of near equal size, but consists of a higher fraction of unknown nucleotides (9.00%). Unless otherwise noted, all simulations were done as haploid genomes; *i.e*., there is only one copy of each genomic sequence. Reads were mapped to their reference with the default values of the long read map program of the CLC Assembly Cell [24]. In essence, then, at least 50% of a read had to share 80% or more sequence similarity with its reference for the former to be mapped to a site of the latter. 

### 3.1. The Effects of Repeats on Sequence Assemblies

We begin with a simple example using human chromosome 2, which further documents the negative effects of repeats on sequence assemblies. Repeats are widely recognized as the major source of ambiguity for the assembly of long contigs from the reads of complex genomes (e.g., for humans and other mammals) [2]. This ambiguity becomes particularly acute when dealing with the short reads of next generation sequencing data [9,10,11]. Such short reads often fail to span the repeats, thereby leading to poor assemblies. To reconfirm these effects, 20 times coverage with unpaired reads of length 100 bases were simulated from human chromosome 2, mapped to this reference, and then arranged into contigs by the assembly simulator. This simulation resulted in contigs with an N_50_ of 42,710 bp and with 96.56% coverage of chromosome 2. In contrast to published studies with real data [37], our N_50_ is calculated according to the entire known reference rather than only against its contigs. That is, each site in the reference is annotated by the length of the longest contig that includes the position. In turn, sites that are not covered by any contigs are assigned zero. The N_50_ is then calculated as the median of the annotated scores for all sites. Given the availability of the reference genome, this modified definition of N_50_ is preferred, even though its values may be slightly inflated due to some short overlaps between neighboring contigs.

As 2.16% of chromosome 2 is unknown, the 96.56% coverage reported above actually corresponds to a contig assembly of the known regions of 98.69%. For comparison, we next generated a completely random sequence with the same length as chromosome 2 and with equal 25% base frequencies. The same read and assembly simulations as with human chromosome 2 (above) were then completed (*i.e.*, 20 times coverage with unpaired read lengths of 100 bases). The results for this random reference are strikingly better than those for human chromosome 2. Three contigs are assembled for the random reference with the length of the longest and its N_50_ equal to 167,886,297 bp. The reason for this remarkably better assembly is that the random reference lacks the numerous repeats that characterize chromosome 2, as well as the rest of the human genome. A few extra contigs of chromosome 2 are due to its 17 unknown regions of at least 10 kbp, but this source of ambiguity is negligible relative to that introduced by the repeats.

### 3.2. Read Length and Coverage

**Figure 6:**
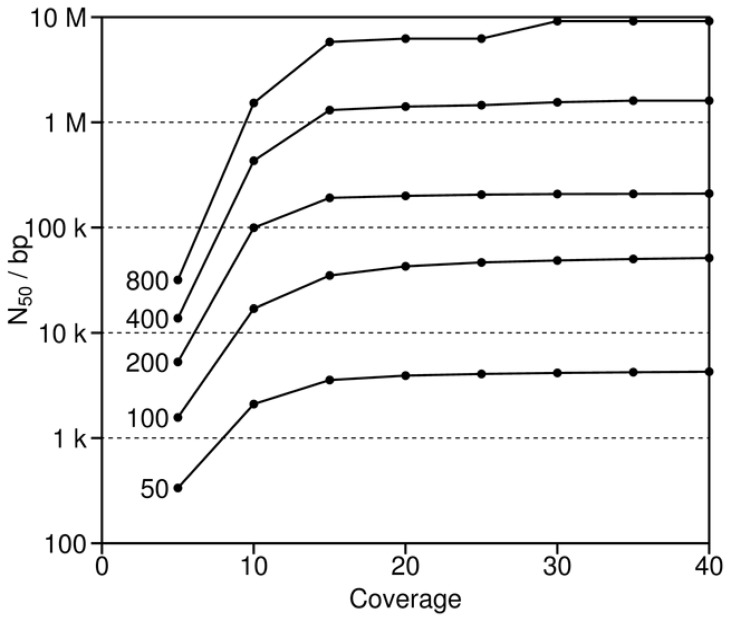
N_50_ as a function of both read length (50 to 800 bases) and coverage. These simulations are based on human chromosome 2.

As also implied by the previous simulations with short reads, long reads are of known value in the generation of good assemblies [38]. Similarly, good coverage is also important. Long reads (or paired end reads, see below) serve to straddle repeats, thereby leading to the assembly of longer contigs. Good coverage reduces the extent of unknown regions and helps to ensure that the overlaps between successive reads are sufficiently long to span the repeats. To study the effects of these two variables more closely, reads of varying fixed length and sequence depth were simulated and then assembled with human chromosome 2 (Figure 6). These simulations show that increasing depth improves the assemblies (as measured by N_50_), but only up to about 15 to 20 times coverage [39]. Thereafter, N_50_ for the various simulations approach different asymptotes that are directly related to increasing read length. The asymptotes for the short reads of 50 and 100 bases are at least 10 to 100 times less than those for the longest reads of 400 and 800 nucleotides. The short reads are characteristic of those for many next generation sequencing projects [9,10,11]. In contrast, the longest reads are not presently feasible, given current sequencing technology, and thus represent instead a theoretical “what if.” In the end, the simulations identify the intermediate reads of 200 bases as of greatest potential interest, since they combine the advantages of the shortest and longest reads. That is, such intermediate reads combine the current feasibility of these data for sequencing projects with the sufficient length to span repeats, which is critical for good assemblies.

Our simulations suggest that sequencing depths of greater than 15 to 20 times coverage provide relatively little return, regardless of the read length (Figure 6). However, this cutoff of 15 to 20 times is most likely too low for real data for a number of reasons. First, the capacity to clone and sequence different regions is not equal due to, e.g., genome wide differences in GC content [40]. Thus, a greater overall depth is needed to ensure that the difficult regions to clone and sequence are also sufficiently covered. Second, greater coverage is required to assemble both haplotypes and homologs of diploid individuals. Third, reads will contain errors that can negatively affect the assembly, particularly when they are short and assembled with De Bruijn graph algorithms (see next section).

### 3.3. Word Size for De Bruijn Graph Assemblers

De Bruijn graph algorithms are vulnerable to particular negative effects of sequencing errors and short reads on their chosen word size [31]. To test for these effects, assemblies were generated with the real reads from the Yoruba individual that was sequenced with the Illumina Genome Analyzer [7]. The sequencing of this human genome was entirely done with paired reads spanning about 200 bp. The reads were of length 36, 37, and 41 bases (89.0%, 7.4%, and 3.6% of the total dataset, respectively). This dataset was reduced for the current assemblies from 42.3 times to 20 times coverage by discarding an appropriate number of randomly chosen pairs. The reads were then treated as unpaired, as well as paired, to test for the benefits of retaining the latter. 

**Figure 7:**
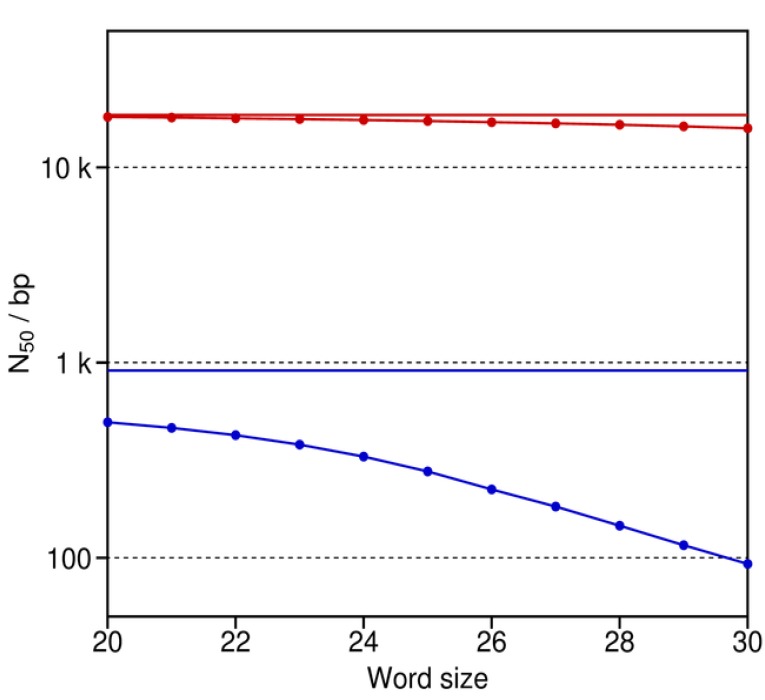
N_50_ as a function of word size for the assembly experiments with the De Bruijn graph approach and the short Illumina reads of the Yoruba individual. The upper (red) and lower (blue) curves are for the De Bruijn graph assemblies with paired *versus* unpaired reads, respectively. The two solid horizontal lines refer to N_50_ for the two optimal assemblies as obtained by our assembly simulator with the paired and unpaired reads. These N_50_ are consistently greater than those for the De Bruijn graph assembler, since our simulator forms contigs against the known reference. Thus, our simulator avoids the use of words and word sizes, thereby minimizing the negative effects of the sequencing errors and short reads in this real dataset.

N_50_ for the unpaired reads shows an obvious decrease in the assembly quality of the De Bruijn graph approach as word size increases, particularly for words of length 25 bases or greater (Figure 7). For many assembly projects of complex genomes, relatively long words of e.g., 27 bases are generally regarded as best [37]. However, the simulations show that the use of both short unpaired reads and long words in a De Bruijn graph assembly comes with a considerable cost. In contrast, the results indicate that this cost is minimal when working with mate pairs, even when their reads are short. The reason is that the paired reads basically function like the long reads in our previous simulations (Figure 6).

Figure 7 illustrates the negative effects of sequencing errors and longer word sizes of 20 or more on sequence assemblies with the De Bruijn graph approach. Conversely, as noted before, the use of shorter words introduces a different set of problems that can limit the quality of the De Bruijn assembly. Specifically, shorter words allow for more connections within the graph, thereby making it very complicated and ambiguous. Thus, the red and blue curves in Figure 7 would also fall off to the left with decreasing word size for real De Bruijn assembly programs. Correspondingly, an optimal word size is expected for them. In contrast, the plots for our assembly simulator will not fall off to the left. The reason is that the ambiguities and complexities of De Bruijn graphs impose limitations on the implementations of real assembly algorithms. Such limitations are not an issue for an ideal De Bruijn assembler (*i.e*., one that is not constrained by its implementation) or our assembly simulator (which relies instead on the available reference for its assemblies).

### 3.4. Sequencing Strategies

Whole genome shotgun sequencing is now the standard for high throughput sequencing and large-scale *de novo* assembly projects [2]. However, alternative sequencing strategies also exist to complete such large scale studies. These different strategies can be compared against each other by our simulation tools to learn more about their relative advantages. One alternative strategy is to first sort the chromosomes by, e.g.. flow cytometry, and then to sequence them individually [14]. The idea here is that the separate handling of chromosomes reduces the number of repeats per chromosome. This chromosome-by-chromosome approach allows for easier assembly of the reads as the negative effects of the repeats are thinned out.

**Figure 8:**
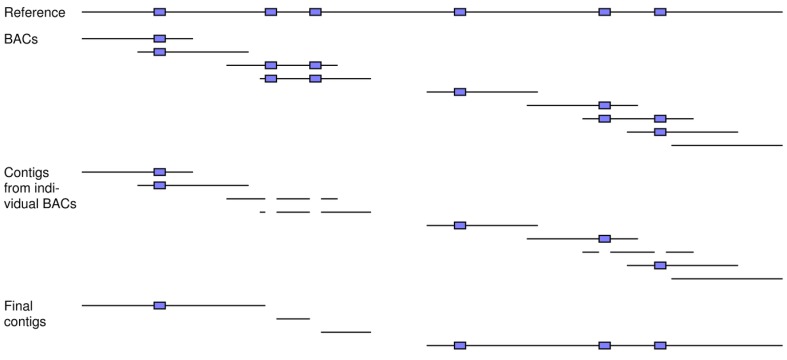
How BAC sequencing can achieve better assemblies and can be modeled by our simulation tools. A number of BAC clones is simulated from the reference, which includes six repeats (blue boxes). A sufficient coverage of reads is generated for each fragment and then assembled into contigs on a clone-by-clone basis. These contigs are next assembled across the clones to generate the final ones for the full set of reads. In this example, clones can carry single copies of the repeats, thereby allowing for unambiguous assembly of their reads into contigs. This divide-and-conquer approach results in greater resolution for the final across-clone assembly of these contigs.

Another available strategy is BAC sequencing, where the long inserts of individual BAC clones (e.g., 150 to 250 kbp) are first separately sequenced [1]. These reads are then assembled into contigs on a clone-by-clone basis (Figure 8). The benefits of this “divide and conquer” approach are similar to those for chromosome sorting. That is, each BAC clone is most likely to carry only a fraction of the repeats in the entire genome, thereby simplifying the assembly of its reads. Once the assemblies of each BAC clone are finished, an assembly of their separate contigs is done to generate the final set for the full genome.

BAC sequencing can be simulated with our simulation tools as follows: (1) A number of BAC fragments of a certain length is simulated from the reference genome; (2) A specified number of reads of a predetermined length is generated from each BAC fragment; (3) The reads of each BAC clone are assembled into contigs; and (4) The contigs of the different BAC clones are assembled together to form the final set for the entire genome. The first and second steps invoke our read simulator, whereas the third and fourth involve our assembly simulator. In steps (3) and (4), each set of reads is first mapped to its BAC fragment and then the resultant contigs for each clone are assembled together against the reference, respectively.

An important extension of BAC sequencing is BAC pooling [41,42]. In BAC pooling, a number of BAC clones (e.g., 100 to 150) is sequenced together rather than one at a time. This strategy is particularly attractive for high throughput (next generation) sequencing technologies, where each channel of the sequencing instrument produces a very large number of short reads [43]. By combining many clones together, each sequencing channel can provide good depth of the different genomic regions that are represented by the various BACs of the pool. However, as the pool only covers a fraction of the genome, this strategy still benefits from a reduction in repeats as do chromosome sorting and BAC sequencing.

This basic BAC pooling approach can be extended even further by the addition of barcodes or tags (*i.e.*, specific short sequences) to the different samples of a pool before they are sequenced [44,45]. In this way, the different reads from a single run of a next generation sequencer can be traced back to their individual BAC clones. Such tracking allows for an even greater subdivision of the repeats, and thereby, further reduction of their negative effects on a sequence assembly.

The assembly simulation results for four alternative sequencing strategies (with the full human genome as the reference) are compared in Table 1. Chromosome sorting results in moderate N_50_ increases of about 50% relative to those for shotgun sequencing. In agreement with Figure 6, only small gains in N_50_ are achieved by both strategies as the overall depth is increased from 25 to 100 times. In contrast, the results for BAC pooling vary considerably with overall coverage [46]. BAC pooling performs about as well as shotgun sequencing when the overall depth is 25 times, but outperforms both this strategy and chromosome sorting when the coverage is increased to 100 times. The reason for these differences is that BAC pooling samples the genome in two successive ways [1]. This approach first samples the genome via its pool of BAC clones and then according to the read depth of their fragments. These two steps are the source of its advantage in assembly (reduction of repeats), but are also the basis of its need for good coverage at two levels (rather than just one). Thus, a 25-times overall depth due to five-times BAC coverage and five-times read coverage yields worse assemblies than those for shotgun sequencing. In agreement with Figure 6, the five-times read coverage is insufficient to maximize the assembly. However, when the read coverage is increased to 10 times, better assemblies are achieved by both BAC pools, as this depth approaches their asymptote for N_50_.

**Table 1:** Comparison of the assembly simulation results for four alternative sequencing strategies. Unpaired reads of length 400 bases were simulated and assembled against the full human genome as the reference. The simulations were performed with both 25- and 100-times overall coverage. The two BAC pools consisted of 1000 or 100 BAC clones. The BAC pools with 25-times overall depth were done with five-times BAC coverage of the genome and five-times read coverage of each clone. The pools with 100-times overall depth were done with 10-times BAC coverage and 10-times read coverage.

	N_50_ / bp		
Strategy	25 times coverage	100 times coverage	
Shotgun sequencing	989,207	1,118,054	
Chromosome sorting	1,460,637	1,648,099	
BAC pooling: 1000 clones	674,853	2,755,538	
BAC pooling: 100 clones	871,681	6,419,123	

### 3.5. Phase Determination

The performance of six different sequencing strategies to generate contigs for phase determination is summarized in Table 2. These assembly simulations for phase determination were based on two copies of chromosome 2 as the reference. Heterozygous sites were modeled as single base differences that occurred with one chance in a thousand at every nucleotide. This rate of 0.1% approximates the estimated heterozygosity per site for the diploid human genome [34]. Among the three shotgun strategies, the unpaired reads of length 400 bases and paired reads with intervening distances between 1500 to 2500 bp perform the worst. The former reads are too short to span multiple heterozygous sites, whereas the latter depends on the unlikely event that the short ends (100 bases) of each mate pair capture a different polymorphic position. The unpaired reads of 2000 bases work best among these three, since their longer lengths are more likely to encompass neighboring heterozygous positions.

In contrast, BAC pooling with either unpaired or paired reads works much better (Table 2). The reason is that two clones for the same region of both homologs are unlikely to co-occur by chance within the same BAC pool [46]. Thus, in effect, BAC pooling samples and sequences the genome as haploid. This effect increases the odds that the reads of a given length will capture multiple heterozygous sites.

**Table 2:** Phase determinations as inferred from the simulated assemblies for six alternative sequencing strategies. The greater N_50_ and % sequenced fractions identify those strategies with longer contigs and thereby better phase determinations (Figure 4). All simulations were done with two copies of chromosome 2. Heterozygous sites were modeled as single base differences with a rate of 0.1% per site. The unpaired reads were of length 400 or 2000 bases. The reads of the mate pairs were 100 bases long with intervening distances from a normal distribution between 1500 and 2500 bp. All shotgun simulations were performed with 50-times total depth (*i.e.*, 25-times coverage for each homolog of this diploid). Each BAC pooling consisted of 243 pools with 100 clones apiece. This pooling resulted in 10-times BAC coverage of the two reference homologs and five-times read coverage of each clone for the same overall depth of 50 times.

Strategy	Data		N_50_ / bp	Sequenced fraction	
Shotgun sequencing	400-base unpaired reads	734		52.47%	
	2000-base unpaired reads	9,311		95.65%	
	2 x 100-base paired reads	2,274		57.66%	
BAC pooling	400-base unpaired reads	384,621		97.03%	
	2000-base unpaired reads	1,594,313		97.17%	
	2 x 100-base paired reads	2,932,847		97.17%	

## 4. Discussion

In a *de novo* sequencing project, an investigator must balance the desired overall quality of the final result with the time and financial costs of the work. The exact nature of this tradeoff will vary according to the size and complexity of the problem with the large repeat-rich genomes of, e.g. mammals, requiring the most effort and expense. As a consequence, researchers must make various decisions as to which available sequencing technology, hierarchical approach, and assembly algorithm are most appropriate for their problem at hand. Our new computer program is provided to assist the researcher in making these decisions in an informed and effective manner. By learning what is theoretically possible for the final result, an investigator may then weigh the different experimental and computational tradeoffs to decide what is realistically best for her or his project. 

Our current simulations reconfirm the value of paired reads and BAC pooling, while documenting once again the central importance of longer read lengths (Figure 6 and Figure 1, and Table 1). Such benefits can now be weighed against the cost of the additional laboratory work to generate the BAC libraries and pools [1,15,44] and in light of the fact that available next-generation sequencers produce short reads of about 35 to upwards of 330 bases [9,10,11]. Our simulations also reconfirm the need for good sequence coverage, but only up to a point after which the return becomes minimal (Figure 6). Furthermore, these results highlight a new approach for sequence assembly, which is of potential utility in the study of phase determination (Figure 4 and Table 2).

In the same vein, we anticipate that our computer program will assist in the further development of *de novo* assembly algorithms. As implementations of the De Bruijn graph approach, current *de novo* assemblers are sensitive to sequencing errors that introduce a cost on their use of longer words (Figure 5 and Figure 7). By controlling the error rate and word size, a developer can use our program to study systematically how these two interacting factors influence the performance of a De Bruijn assembly algorithm. For example, one can use our read simulator to generate reads of varying length and with specific rates and types of sequencing errors to study the optimal word sizes for different assembly programs. Such studies will offer insights as to how these factors limit different implementations of the De Bruijn graph approach.

The above simulations, comparisons, and possibilities highlight just some of the many ways that our new program can contribute to the further study of *de novo* sequence assembly. Others that we wish to emphasize here include a systematic comparison of how varying BAC coverage *versus* read depth per clone can improve the BAC pooling approach. For example, our use of five-times BAC coverage *versus* five-times read coverage per clone (for an overall depth of 25 times) resulted in poor assemblies for the basic BAC pooling approach (Table 1). Would changing the depths to, e.g. 2.5-times BAC coverage and 12-times read coverage (for the same overall depth of 25 times), have helped in this case? Our computer program simulates a number of different kinds of sequencing errors, but not those due to chimeric sequences, premature stops, and cloning biases [40,47]. In particular, chimeric sequences that can form during DNA cloning and amplification remain of special concern for those using paired end reads and/or studying phase determination. Ultimately, as emphasized throughout, our optimal assemblies should be compared against those for available assembly algorithms. Such comparisons will permit the field to explore new approaches and to refine current ones, thereby leading to better strategies and methods for *de novo* assembly. Conversely, we also expect that much will be learned from these comparisons about our new program. For example, such comparisons will provide reality checks on its performance and allow for assessments as to exactly how much and in what ways it benefits from reference to a known standard. 

## 5. Program Availability

The software for our new computer program (read and assembly simulators) is freely available in binary form from CLC bio [23]. Our new program is written in C. It is accompanied by a set of instructions on its use, example input files, and a translation tool for converting datasets in the common SAM format of many read mapping programs into that of the CLC Assembly Cell [24]. Although not a *de novo* assembler, our program (like them) becomes very memory and CPU intensive when dealing with the huge datasets for complex genomes. For example, our new program required 42 GB of memory and a computation time of 6 to 10 hours (depending on the simulation word size and whether the data were paired) for the assembly simulations of the short Illumina reads for the Yoruba individual in Section 3.3. These simulated assemblies were done on a single core of a 2.67 GHz Intel Xeon X5550 CPU. These memory requirements and run times are significantly less than would be needed for a full *de novo* assembly with (e.g.) the Velvet program [16].
